# The prospective mathematical idea satisfying both radiation hormesis under low radiation doses and linear non-threshold theory under high radiation doses

**DOI:** 10.1186/s41021-020-0145-4

**Published:** 2020-02-03

**Authors:** Katsuhito Kino

**Affiliations:** Kagawa School of Pharmaceutical Sciences, Tokushima Bunri University, 1314-1 Shido, Sanuki, Kagawa 769-2193 Japan

**Keywords:** Radiation hormesis, LNT theory, Inhibition effect

## Abstract

It has yet to be determined whether or not the probability of developing cancer due to radiation exposure levels of low doses is proportional to the dose. Herein, for radiation hormesis occurring at low doses, mathematical models using functions that take a mountain-like shape having two inflection points are considered. The following perspectives were obtained: (i) When the probability of developing cancer decreases at radiation levels above the natural background dose, the radiation hormesis effect occurs up to ~ 12.4 mSv. (ii) When there is a proportional relationship at ≥750 mSv, the radiation hormesis effect occurs up to ~ 225 mSv. Thus, by performing studies at the molecular and cellular levels for radiation doses at ≤16.8 or 307 mSv, it is possible to investigate carcinogenesis resulting from low radiation doses.

## Background

As radiation has the ability to ionize substances, the idea that, as far as is possible, it is best to avoid any exposure is generally accepted. As the basis for this, the International Commission on Radiological Protection (ICRP) employs a model whereby the probability of developing cancer is proportional to the radiation exposure dose (Linear non-threshold theory: LNT) [1]. Certainly, a proportional relationship has been described at ≥100 mSv, but it has not been confirmed whether there is a proportional relationship for cases of < 100 mSv [1]. In addition, the radiation hormesis theory is thought to actually have beneficial effects on health [2–10]. Therefore, I wondered if there was an idea that LNT and hormesis could hold at the same time. In this paper, such a mathematical idea is considered.

### Probability of developing cancer *D*(*x*) and the inhibition effect *R*(*x*)

Hereafter, the radiation dose is defined as *x*, and the probability of developing cancer as *D*(*x*). Accepting the fact that at high doses, the probability of developing cancer is proportional to the dose, and taking the constant of proportionality to be *k*, Eq. 1 holds true. Here, for a hormesis region to be present at low doses, an inhibitor factor that reduces *D*(*x*) becomes necessary. The inhibition effect relating to the inhibitor factor is described by *R*(*x*). Taking this inhibition effect into account, *D*(*x*) may be defined as in Eq. 2 [11]. Here, it is assumed that both *D*(*x*) and *R*(*x*) are continuous functions. It should be noted that *D*(*x*) ≥ 0 is always satisfied, since there is no possibility that the probability of developing cancer becomes negative.

1 $$ D(x)= kx $$

2 $$ D(x)= kx-R(x) $$

For actual in vivo cases, there exist many inhibitor factors including DNA repair, removal of active oxygen, and apoptosis. However, compared to the inhibitor factor that has the greatest effect on *D*(*x*), the effects of other inhibitor factors on *D*(*x*) are small or non-existent. Thus, in the following, for the sake of simplicity, a single inhibitor factor to be present is considered.

**Fig. 1:**
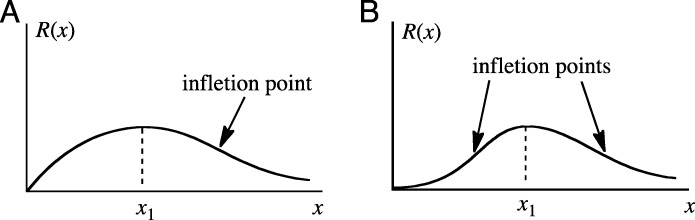
Graph of inhibition effect *R*(*x*) due to the inhibitor factor. The radiation dose is defined as *x*. The radiation dose at which *R*(*x*) reaches a maximum is defined as *x*_1_*.* **a** Single point of inflection for *x* > *x*_1_. **b** One single point of inflection for 0 < *x* < *x*_1_ and another for *x* > *x*_1_

Next, the form of *R*(*x*) is considered. Assuming the inhibitor factor is triggered only by radiation, when *x* is 0 mSv the value of *R* must be 0 (*R*(0) = 0). As the radiation dose increases, *R*(*x*) increases, but if the radiation increases excessively, the inhibitor factor itself becomes inactivated by the radiation, and thus *R*(*x*) begins to decrease at a certain dose. As a result, as *x* approaches infinity, the value of *R* becomes 0. Two forms of graphs for *R*(*x*) having these characteristics can be imagined, and are shown in Fig. 1. The radiation dose at which *R*(*x*) reaches a maximum is defined as *x*_1_*.* The graph in Fig. 1a has a single point of inflection with *x* > *x*_1_. On the other hand, the graph in Fig. 1b has a single point of inflection in each of the regions 0 < *x* < *x*_1_ and *x* > *x*_1_.

**Fig. 2:**
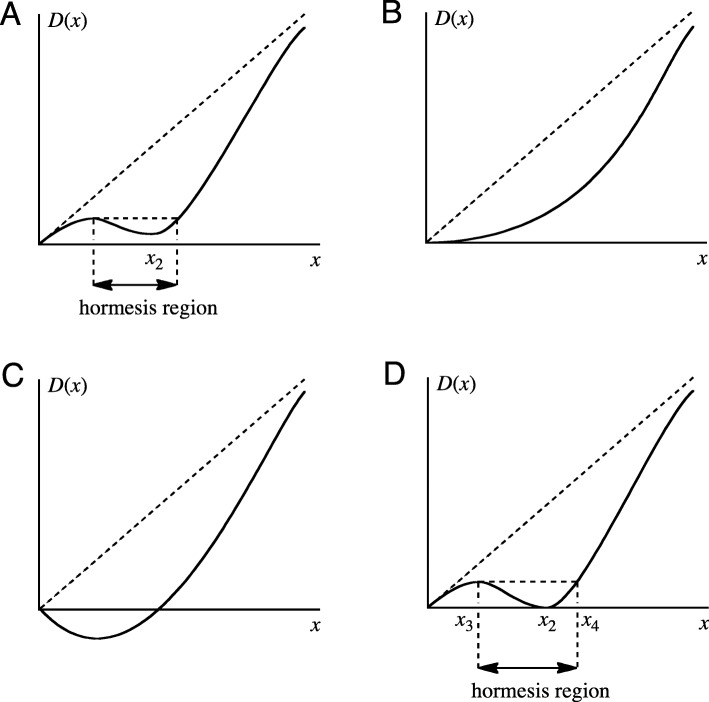
Graph of probability of developing cancer *D*(*x*). The radiation dose is defined as *x*. The linear dashed lines from the zero points indicate LNT (Eq. 1). **a** *D*(*x*) with hormesis region present. The value of *x* for the local minimum of *D*(*x*) is defined as *x*_2_, for which Eq. 6 must be satisfied. **b** *D*(*x*) with no hormesis region present. **c** *D*(*x*) having negative values. **d** *D*(*x*) of type **a**. having the maximum hormesis region. The value of *x* for the local minimum of *D*(*x*) is defined as *x*_2_, for which Eqs. 5 and 6 must be satisfied. The value of *x* for the local maximum of *D*(*x*) is defined as *x*_3_, and the other value of *x* where *D* has the same value as the local maximum *D*(*x*_3_) is defined as *x*_4_ (ZEP). The hormesis region is *x*_3_ < *x* < *x*_4_

When *D*(*x*) has a hormesis region for *x* > 0, the graph of *D*(*x*) has the form shown in Fig. 2a. For the case where *R*(*x*) takes the form in Fig. 1a, *D*(*x*) takes the form in Fig. 2b or c. Fig. 2b clearly has no hormesis region. Furthermore, although Fig. 2c has a hormesis region, it does not fulfill the condition that *D*(*x*) ≥ 0. Thus, in order for *D*(*x*) to have the form shown in Fig. 2a, it is necessary for *R*(*x*) to have the form shown in Fig. 1b.

In this paper, for *R*(*x*) to have the form shown in Fig. 1b, i chose the simple function given in Eq. 3 among several functions. The constant *a* is understandably positive. The value of *x*_1_ in Eq. 3 is 2*/a*, so *x*_1_ depends solely on the constant *a*. By varying *a*, the radiation dose *x*_1_ where the inhibitor amount reaches a maximum can be freely changed, and *R*(*x*) can be freely adjusted with respect to *D*(*x*).

3 $$ R(x)={x}^2{e}^{- ax}\kern0.5em \left(a>0\right) $$

4 $$ D(x)= kx-{x}^2{e}^{- ax} $$

Substituting Eq. 3 into Eq. 2, Eq. 4 is obtained. *D*(*x*) is a continuous function, and when it has the maximum hormesis region based on fulfillment of the condition *D*(*x*) ≥ 0, it has the form seen in Fig. 2d. The value of *x* for the local minimum of *D*(*x*) is defined as *x*_2_, for which Eqs. 5 and 6 must be satisfied. Then, *x*_2_ = 1*/a* and *ka* = 1/*e* are obtained.

5 $$ D\left({x}_2\right)=0 $$

6 $$ \frac{dD\left({x}_2\right)}{dx}=0 $$

The value of *x* for the local maximum of *D*(*x*) is defined as *x*_3_, and the other value of *x* where *D* has the same value as the local maximum *D*(*x*_3_) is defined as *x*_4_. Previously, *x*_4_ has been defined as being the zero equivalent point (ZEP) [3]. As *x*_3_ and *x*_4_ cannot be solved analytically, approximate values were obtained through numerical calculations using the graphing calculator “Grapher 2.5” as *x*_3_ = ~ 0.285/*a* and *x*_4_ = ~ 1.469/*a*. It is clear that *x*_2_, *x*_3_, and *x*_4_ do not depend on *k*, and *x*_4_ is ~ 5.15 times greater than *x*_3_.

The radiation hormesis effect posits that the probability of developing cancer decreases at radiation levels above the natural background dose. Therefore, up to a certain dose above the natural background, *D*(*x*) should decrease. Thus, the natural background radiation dose can be taken to be between *x*_3_ and *x*_2_. In order to maximize the hormesis region, *x*_3_ is set to the natural background dose, and then ZEP extends to up to ~ 5.15 times the value of the natural background dose. In addition, it is noted that the multiple, 5.15, is independent of the constant *a*. When the worldwide average dose of the natural background radiation is taken to be 2.4 mSv [12], the maximum ZEP is ~ 12.4 mSv.

Furthermore, as 2.4 mSv is *x*_3_ = ~ 0.285/*a*, the value of *x* corresponding to 100 mSv (*x*_5_) becomes ~ 11.875/*a*. Here, it is considered whether Eq. 4 can be approximated to Eq. 1 at 100 mSv. Changing the form of Eq. 4 into that of Eq. 7 and substituting *ka* = 1/*e*, Eq. 8 is obtained. Furthermore, by substituting *x*_5_ *=* ~ 11.875/*a*, Eq. 9 is obtained. At 100 mSv and above, Eq. 4 satisfied by *ka* = 1/*e* can be approximated to Eq. 1. Thus, taking Eq. 4 as the model, linearity is satisfied above 100 mSv.

Lastly, when ZEP is ~ 12.4 mSv, *x*_1_ for the maximum of *R*(*x*) becomes ~ 16.8 mSv. That is, the inhibitor factor of which the amount is a maximum at ~ 16.8 mSv may show the maximum hormesis effect.

7 $$ D(x)= kx\left(1-\frac{x{e}^{- ax}}{k}\right) $$

8 $$ D(x)= kx\left(1- ax{e}^{1- ax}\right) $$

9 $$ D\left({x}_5\right)=k{x}_5\left(1-11.875{e}^{-10.875}\right)\approx 0.9998k{x}_5 $$

### Maximum hormesis region when the *x*^*2*^ term in Eq. 3 is replaced with an *x*^*n*^ term

If the *x*^*2*^ term in Eq. 3 is replaced with an *x*^*n*^ term, a graph of the form of Fig. 1b can still be achieved. That is, Eq. 10 is expressed in place of Eq. 4. Taking the case of the maximum hormesis region, Eqs. 5 and 6 must be satisfied. Therefore, *x*_2_ = (*n*–1)/*a* and *ka*^*n*–1^ = {(*n*–1)/*e*}^*n*–1^ are obtained.

10 $$ D(x)= kx-{x}^n{e}^{- ax} $$

Since the case when *n* = 2 has already been considered in the second section, the cases where *n* ≥ 3 will be considered in sequence. As these cases are also impossible to solve analytically, the solutions are determined using numerical calculations. For *n* = 3, 4, 5, and 10, the maximum ZEP is ~ 10.1, ~ 8.9, ~ 8.1, and ~ 6.3 mSv, respectively. That is to say, as *n* increases, the maximum ZEP becomes smaller. Thus, Eq. 4 (Eq. 10 when *n* = 2) is best suited to consider the maximum hormesis region.

### Condition for a hormesis region to be present for Eq. 4

**Fig. 3:**
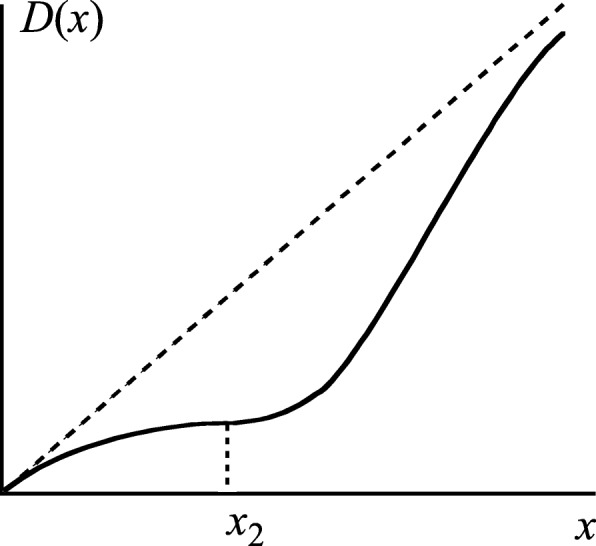
Probability of developing cancer *D*(*x*) at the point where a hormesis region begins to appear. The radiation dose is defined as *x*. The value of *x* is defined as *x*_2_, for which Eqs. 11 and 12 must be satisfied. The linear dashed line from the zero point indicates LNT (Eq. 1)

The condition for *ka* giving the maximum hormesis region was considered in the second section. In contrast, in this section, the condition for *ka* under which the hormesis region begins to appear (Fig. 3) is determined.

The condition for which the hormesis region begins to appear is given by Eqs. 11 and 12, and then ${x}_2=\left(2-\sqrt{2}\right)/\mathrm{a}$ and $ka=2\left(\sqrt{2}-1\right){e}^{\sqrt{2}-2}$ were determined. Therefore, when combined with the conclusions of the second section, the condition for which the hormesis region appears is 1/*e* ≤ *ka* < $2\left(\sqrt{2}-1\right){e}^{\sqrt{2}-2}$. Expressed to three significant digits, this corresponds to ~ 0.368 ≤ *ka* < ~ 0.461. Thus, for Eq. 4 to have a hormesis region, the restrictive condition must be fulfilled.

11 $$ \frac{dD(x)}{dx}=0 $$

12 $$ \frac{d^2D(x)}{d{x}^2}=0 $$

### Reconsidering the second section when there is a proportional relationship at ≥750 mSv

Siegel et al. asserted that threshold is 0.75 Gy [13]. Reconsidering the assumption that *x*_3_ is the natural background radiation dose in the second section 2, the condition by which a proportional relationship should approximately hold at ≥750 mSv and accepting a ≤ 10% error is imposed. Using Eq. 8, it is necessary for Eq. 13 to be satisfied. Thus, *x*_6_ corresponding to 750 mSv was determined to be ~ 4.890/*a*. From *x*_6_, it was determined that *x*_3_ = ~ 0.285/*a* and *x*_4_ = ~ 1.469/*a* correspond respectively to ~ 43.7 and ~ 225 mSv. Summarizing the results of the above calculations, when satisfying the proportional relationship with an error within 10% at ≥750 mSv, the maximum hormesis region becomes 43.7–225 mSv. In addition, *x*_1_ for the maximum of *R*(*x*) becomes ~ 307 mSv.

13 $$ D\left({x}_6\right)=k{x}_6\left(1-a{x}_6{e}^{1-a{x}_6}\right)=0.9k{x}_6 $$

### Conclusion and implication

When the probability of developing cancer decreases at radiation levels above the natural background dose, the maximum ZEP becomes ~ 12.4 mSv, and at the same time, a proportional relationship is approximately obtained at ≥100 mSv. At ~ 16.8 mSv, *R*(*x*) reaches a maximum. Additionally, for Eq. 4, a hormesis region appears when ~ 0.368 ≤ *ka* < ~ 0.461.

When there is a proportional relationship at ≥750 mSv, the maximum ZEP becomes ~ 225 mSv. At ~ 307 mSv, *R*(*x*) reaches a maximum.

Since statistically measuring *D*(*x*) at low doses is effectively not possible, analyzing the following three points would help clarify the radiation hormesis effect, perhaps making it possible to determine the probability of developing cancer at low doses.

(i) Finding a factor which expressed inhibition effect versus dose has the approximate form of Fig. 1b.
(ii) Analyzing the variations of the inhibitor factor in the region up to ~ 16.8 or 307 mSv.
(iii) Determining *k*, which indicates the correlation between *D*(*x*) and *R*(*x*).

Although preliminary, it is felt that the results and discussions presented in this paper may be of potential use to other researchers. Furthermore, if such inhibition factors are identified, it might possibly lead to a method of effectively reducing the cancer rates.

## Data Availability

All data generated during this study are included in this published article.

## Abbreviations

Eq: Equation
ICRP: International Commission on Radiological Protection
LNT: Linear non-threshold theory
ZEP: Zero equivalent point

## References

[1] ICRP (2006). Low-dose extrapolation of radiation-related cancer risk. ICRP publication 99. International commission on radiological protection.

[2] Socol Y, Dobrzynski L, Doss M, Feinendegen LE, Janiak MK, Miller ML, Sanders CL, Scott BR, Ulsh B, Vaiserman A (2014). Ethical issues of current health-protection policies on low-dose ionizing radiation. Dose Response.

[3] Luckey TD (1991). Radiation hormesis.

[4] Sanders CL (2010). Radiation hormesis and the linear-no-threshold assumption.

[5] Feinendegen LE, Bond VP, Sondhaus CA. The dual response to low-dose irradiation: induction vs. prevention of DNA damage. In: Yamada T, Mothersill C, Michael BD, Potten CS. Biological effects of low dose radiation. Excerpta Medica. International Congress Series 1211. Amsterdam: Elsevier; 2000, p. 3–17.

[6] Sutou S (2016). A message to Fukushima: nothing to fear but fear itself. Genes Environ.

[7] Sutou S (2017). The 10th anniversary of the publication of genes and environment: memoir of establishing the Japanese environmental mutagen society and a proposal for a new collaborative study on mutagenic hormesis. Genes Environ.

[8] Sutou S (2018). Low-dose radiation from A-bombs elongated lifespan and reduced cancer mortality relative to un-irradiated individuals. Genes Environ.

[9] Sutou S, Koeda A, Komatsu K, Shiragiku T, Seki H, Yamakage K, Niitsuma T, Kudo T, Wakata A (2018). Collaborative study of thresholds for mutagens: proposal of a typical protocol for detection of hormetic responses in cytotoxicity tests. Genes Environ.

[10] Rattan SIS, Bourg EL (2014). Hormesis in health and disease.

[11] Kiefer J (1990). Biological radiation effect.

[12] UNSCEAR (2008). Sources and effects of ionizing radiation. UNSCEAR 2008 Report.

[13] Siegel JA, Sacks B, Socol Y (2017). The LSS cohort of atomic bomb survivors and LNT. Comments on “Solid Cancer incidence among the life span study of atomic bomb survivors: 1958-2009” (Radiat Res 2017; 187:513-37) and “Reply to the comments by Mortazavi and Doss” (Radiat Res 2017; 188:369-71). Radiat Res.

